# Time to Consider Moving Beyond Exclusive Breastfeeding in Southern Africa

**DOI:** 10.3390/children4010007

**Published:** 2017-01-24

**Authors:** Janet M. Wojcicki

**Affiliations:** Department of Pediatrics, University of California, 550 16th Street, San Francisco, CA 94158, USA; janet.wojcicki@ucsf.edu

**Keywords:** Sub-Saharan Africa, mother to child transmission, mixed feeding, breastfeeding, prevention of mother to child transmission

## Abstract

While there have been considerable advances in the reduction of mother to child transmission of HIV (MTCT) in sub-Saharan Africa with the advance of anti-retroviral therapies (ART), there remain challenges in the late postpartum period.  Structural issues including food insecurity and stigma make better maternal ART adherence and exclusive breastfeeding unreachable for some women. There are no other scientifically researched feeding options as there have been few studies on different types of mixed feeding practices and risk of HIV infection. Additional studies are warranted to assess detailed feeding practices in HIV exposed infants in relation to clinical outcomes.

## 1. Low Prevalence of Exclusive Breastfeeding in Resource-Poor Settings

Although exclusive breastfeeding is widely understood to have a number of significant benefits for infant and child health, low- and middle-income countries continue to have low prevalence of exclusive breastfeeding rates [1]. A recent review from the *Lancet* on breastfeeding in the 21st century estimated that only 37% of children under 6 months of age are exclusively breastfed in low- and middle-income countries [1]. Exclusive breastfeeding rates are even lower for Southern Africa; for example, only 7% of infants are exclusively breastfed in Swaziland, along with only 11% and 14% of infants in Zambia and Lesotho, respectively.

In particular, human immunodeficiency virus (HIV)-exposed children—who comprise a large group of children overall in Southern Africa—have an increased risk of serious infections and mortality if they are not breastfed, especially in the first three months of life. Furthermore, in the context of HIV transmission, numerous studies in South Africa have found that exclusive breastfeeding reduces HIV transmission by approximately 52%, and therefore exclusive breastfeeding serves as a higher protection against viral transmission versus mixed-feeding (mixing breastmilk with liquids and solids) [2]. 

However, in spite of huge gains made in pregnancy, labor, delivery and the early postpartum period by widespread ART usage, along with high reductions of mother-to-child transmission (MTCT)—close to 5% in some areas [3]—by 18 months of age, the cumulative incidence of HIV in infants can be between 10%–15% in Swaziland and other areas of the Southern African region [4]. This high cumulative incidence of infection by 18 months of age can most likely be explained by low maternal adherence to ART under Prevention of Mother-to-Child Transmission (PMTCT) programs, along with a low percentage of women exclusively breastfeeding until six months of age.

## 2. Targeted Interventions for Women Who Will Not Exclusively Breastfeed or with Limited Antiretroviral Therapy (ART) Adherence

Public health workers need to first assess the failures and limitations of past efforts to increase exclusive breastfeeding rates and subsequently answer the question of whether women with certain risk factors for poor adherence to ART and/or are intent on using mixed-feeding, should be provided other options instead. Recent studies of adherence to option B+ (ART for life for infected women) suggest that adherence was adequate for only 66% of women in the first three months postpartum and only 30% of women maintained adequate adherence at every visit [5].

Counseling for exclusive breastfeeding is labor intensive and costly and structural barriers to exclusive breastfeeding may not easily be remedied in spite of additional interventions. Recent data in Swaziland suggest that only 56%, 40% and 21% of eligible mothers picked up ART refills atone, two and three months, respectively, and postpartum retention was only 50% for women on Option B+ [6,7]. Similar rates of loss during the postpartum period are reported in South Africa, with 49% of women having missed a postpartum visit or became disengaged from care by 6 months [8]. Structural factors, including food insecurity, stigma in the community, financial constraints, distance to the clinic and long waiting times can greatly impact retention and adherence to PMTCT services. In order to reduce mother-to-child transmission to the lowest possible levels, it may also be necessary to recognize that there are other feeding alternatives to exclusive breastfeeding, which minimize risk and are similarly associated with HIV transmission rates similar to exclusive breastfeeding.

Our understanding of choices and feeding patterns of women in Southern Africa who use mixed-feed their infants is currently limited. Further studies are needed, especially in the context of Option B+.

## 3. Need to Better Understand Mixed-Feeding Patterns

A couple of earlier studies suggest that solids may pose additional risks for HIV infection in exposed infants compared to mixed-feeding with liquids alone. The large and complex proteins found in solid foods may cause a greater amount of damage to the gastrointestinal (GI) mucosa or regulate the gut mucosa differently. These studies have suggested that prior to six months of age, there is an increased risk for solid food consumption, in comparison to infants fed only liquids [9,10]. A study from South Africa and Western Africa found that infants who were introduced solids in the first two months of life were 2.9 (1.1–8.0) times more likely to be infected postnatally than those who had never been exposed to solids but only exclusively breastfed or mixed-fed with other liquids [9]. Similarly, a study from South Africa by Coovadia et al. [10] found that infants receiving solids in the first six months of life were 11 times more likely to get infected with HIV than those receiving breast milk alone. While these studies were conducted in the pre-ART era, the gut inflammation that occurs from exposure to solids most likely happens independently of maternal HIV suppression.

More recent studies, in the context of ART availability, also suggest that mixed feeding versus exclusive breastfeeding is associated with greater risk of mother-to-child transmission of HIV. However, these studies do not differentiate between solid versus non-breast-milk liquids in determining increased risk, and therefore further research need to be done to elucidate this difference [11].

Mixed-feeding is common, with complementary foods often introduced too early in Southern Africa, and yet we know very little about the types of foods introduced and the relationship with HIV infection. Ethnographic research from South Africa, Namibia and Swaziland found that even though mothers knew that nurses discouraged mixed-feeding, many would give infants water daily and complementary feeding as early as ten days of age, believing that breast milk alone was insufficient to feed a child [12]. Recent data from Rwanda suggest that even in the context of Option B+ monthly clinic visits, exclusive breastfeeding rates can drop off by 6 months of age, with less than 50% of women still exclusively breastfeeding [13].

## 4. Recommendations for the Future

While governments, non-profit organizations and public health workers continue to direct interventions to improve exclusive breastfeeding rates in high-risk populations, recognition of the limitations of these efforts should also be taken into consideration for specific population groups. Furthermore, gaps in our understanding of patterns of mixed-feeding in high-risk populations should be addressed, as it is reasonable to suggest that there may be differential risk associated with different types of mixed-feeding. Specifically, additional studies may help elucidate particularly risky behavior, such as the early intervention of solids versus non-milk liquids, which could subsequently be targeted by healthcare workers for at-risk population groups.
